# Editorial

**Published:** 1838-12-05

**Authors:** 


### THE MEDICAL EXAMINER.

PHILADELPHIA, DEC. 5, 1838.

### INSANE HOSPITAL.

A highly respectable and large meeting of citizens of Philadelphia, was held at the Supreme Court Room, on Thursday the 29th of November, to request the aid of the Legislature in erecting hospitals for the insane poor of the state. We have, for some years, been convinced of the necessity of some such measure. From an official connexion with the only hospital in which the greater part of the insane poor of the city and county are admitted, we have ascertained, most completely, that no adequate provision for the management of chronic cases of insanity can be made at an hospital which is not exclusively destined for this purpose.

The Pennsylvania Hospital, for the insane, is a large institution at a little distance from the city, and, when completed, will, doubtless, present all the advantages of the best conducted institutions. The insane, who are now treated in a building not entirely adapted to their use, will then enjoy the advantages of air and exercise, with the restorative means afforded by regular employment. We need an institution of the same kind for the poor, and if the attention of the public spirited individuals, who have at present suggested this measure, should be carried out, we have no doubt that an insane hospital will soon be established in Pennsylvania, which will be in nowise inferior to the excellent institutions already so liberally endowed by eleven of the states.

